# Cognitive Health of Nonagenarians in Southern Italy: A Descriptive Analysis from a Cross-Sectional, Home-Based Pilot Study of Exceptional Longevity (Cilento Initiative on Aging Outcomes or CIAO)

**DOI:** 10.3390/medicina56050218

**Published:** 2020-05-05

**Authors:** Vincenzo Pizza, Paola Antonini, Rossella Marino, Giovanni D’Arena, Serena Grazia Lucibello, Marianna Rizzo, David A. Brenner, Dilip V. Jeste, Salvatore Di Somma

**Affiliations:** 1 Great Network, 00191 Rome, Italy; v.pizza@aslsalerno.it (V.P.); paola.antonini@greatnetwork.org (P.A.); salvatore.disomma@greatnetwork.org (R.M.); gidarena@gmail.com (G.D.); serena.lucibello@gmail.com (S.G.L.); nutrizionista.rizzo@gmail.com (M.R.); 2 Department of Emergency and Time Dependent Networks, 84124 ASL Salerno, Italy; 3 Great Health Science, 00100 Rome, Italy; 4 Department of Medicine, University of California San Diego, La Jolla, CA 92093, USA; dbrenner@health.ucsd.edu; 5 Departments of Psychiatry and Neurosciences, and Sam and Rose Stein Institute for Research on Aging, University of California San Diego, La Jolla, CA 92093, USA; djeste@health.ucsd.edu; 6 Department of Medical-Surgery Sciences and Translational Medicine, La Sapienza University, 00189 Rome, Italy; 7 Department of Emergency Medicine, University of California San Diego, La Jolla, CA 92093, USA

**Keywords:** Cilento Region, longevity, cognitive health, lifestyle

## Abstract

*Background and objectives:* Nonagenarians and centenarians (NCs) are an extremely fragile population, particularly in regard to their physical and cognitive function. The aim of this study was to define the neurocognitive profiles among 29 NCs and their 49 younger cohabitants aged 50–75 years from The Cilento Initiative on Aging Outcomes (CIAO) Pilot study in the South of Italy that had provided initial hypotheses regarding positive psychological traits related to exceptional longevity. *Materials and Methods*: During the home visits, lifestyle information with specific questionnaires, functional autonomy and the neuropsychological Mini Mental Scale Examination (MMSE), and the Alzheimer’s Disease Assessment Scale-Cognitive (ADAS-Cog) scale were obtained by qualified study personnel. The total blood oxidative capacity was also determined by testing the reactive derivative of oxygen metabolites (d-ROM) and by the Biological Antioxidant Potential (BAP). In all individuals, the APOE genotype determination was also performed. *Results*: All the subjects in both groups showed high adherence to the Mediterranean Diet. None of the NCs had severe cognitive impairment, and a very low incidence of dementia was found. The data obtained on the Activities ed Instrumental Activities of Daily Living (ADL-IADL) scale showed that the majority of NCs (16/29) were autonomous in daily life activities. The comparative assessment of NCs and cohabitants showed no significant differences in the laboratory assessment of oxidative stress and APOE genotype. *Conclusions:* In the Cilento Region of Southern Italy, NCs seemed to have good cognitive status when compared to younger cohabitants aging 50–65 years without significant differences in oxidative stress markers or APOE genotype. These results might be related to optimal adherence to the Mediterranean diet, although other lifestyle factors and positive personality traits may also contribute to their healthy aging. Further studies on a larger population should be performed to confirm the results of this pilot study.

## 1. Introduction

In Western countries, over the next 30 years, the proportion of older adults (>65 years old) will double from 11% to 22% of the total population [1]. This increase in numbers will be more evident in developed countries, where the number of subjects aged over 80 years will be almost quadrupled [2,3]. The health status of the older population is no longer identified solely by the incidence of diseases, but it is also linked to the maintenance of psychosocial and relational well-being, even in the presence of multi-morbidity. 

Nonagenarians and centenarians (NCs) represent the best model of study for exceptional human longevity [4], although in industrialized countries, they represent a small fraction of the overall population, approximately only 1 in 5000–10,000 [4]. Moreover, even if they are considered an extremely fragile population from physical and cognitive perspectives, the presence of their health conditions is often difficult to assess, particularly in regard to the cognitive function that, in turn, can be affected by sensory deficits. Furthermore, the available cognitive tests are standardized for a population that does not exceed 80–85 years of age in most cases [5]. For the very old population, the definition of physical limitations could also be difficult to categorize, and for this reason, classifications by three different stages (A, B, and C) have been proposed for these subjects using a series of objective parameters [6].

Studies on NCs present a series of limitations such as rarity of the sample, absence of an appropriate age control group, frailties related to advanced age, and difficulty in visiting them in the research settings for a complete medical examination since they generally do not like to move from their own home.

The Cilento Initiative on Aging Outcomes (CIAO) Pilot study was a home-based study initiated to assess the possible impact of various factors on human longevity and age-related diseases. We previously reported the results of this study on psychological traits through qualitative interviews among 29 NCs and their 52 younger cohabitants aged 50–75 years [7], and more recently, we described the cardiovascular profiles of the Cilento nonagenarians [8]. The aim of the present component of the pilot CIAO study was to define the cognitive status and autonomy of NCs, with the eventual goal of developing initial hypotheses on the neurocognitive profile and lifestyles associated with exceptional longevity.

## 2. Materials and Methods

This study is a collaboration involving the La Sapienza University of Rome (Italy), the University of California San Diego (La Jolla, CA, USA), the Waltraut Bergmann Foundation (Berlin, Germany), and the GREAT Research Network (Italy).

### 2.1. Study Sites

This descriptive, community-based, cross-sectional study was conducted over two months, in June and July 2016, at the National Park of Cilento, Vallo di Diano, and Alburni (Salerno, Italy). Subjects living in the following villages were enrolled: Acciaroli, Casal Velino, Gioi, Futani, Montano Antilia, Pattano, San Mauro Cilento, San Mauro La Bruca, Sessa Cilento, Stella Cilento, and Vallo della Lucania. The study was conducted in the homes of enrolled subjects.

### 2.2. Patients and Methods

For this pilot study, we worked in collaboration with local General Practitioners (GPs) who compiled a list of 29 households, each encompassing at least one subject aged ≥90 years and up to two cohabitants aged 50 to 75 years, of either sex, for a total of 78 individuals, of whom 29 were NCs. The selection of the NC and their families was made by Cilento family doctors and was based on a single inclusion criterion—that each household was composed of at least one NC and two cohabitants from 50 to 75 years of which doctors had complete records. All the participants were visited at home by a psychologist, a neuropsychologist, a nutritionist, and a team of cardiologists. The specialists planned their visits to the subjects’ homes so as not to overlap and to have the time necessary for each evaluation.

Blood samples were also collected at home by Central Lab nurses (Lab D’Arena, Vallo della Lucania). The study received approval from the ethics committee of the ASL Salerno (Comitato Etico Campania Sud), and the Human Subjects Protection Committee of the University of California San Diego. All participants provided written, informed consent to participate in the study.

### 2.3. Lifestyle Assessment

Lifestyle information was obtained using questionnaires administered by trained study personnel and focused particularly on dietary lifestyle, physical activity, alcohol consumption, and smoking habits. The dietary lifestyle was assessed by a nutritionist who evaluated the daily food intake at the participants’ homes. Adherence to the traditional Mediterranean diet was assessed using the Mediterranean Diet Score (MDS) index, whose score ranges from 0 (no adherence to the Mediterranean Diet) to 9 (maximal adherence) [9]. Nine food groups or nutrients were considered—fruits, vegetables, legumes, cereals, fish, meat and meat products, milk and dairy products, wine, and extra virgin olive oil. Moreover, a complete visit for clinical judgment from the GP was performed at the same time.

### 2.4. Neurological and Psychometric Assessments

The neuropsychological and functional autonomy tests utilized in our study for the evaluation of the cognitive status of NCs and their cohabitants were MMSE (Mini-Mental State Examination) [10,11] in all subjects, while ADAS-Cog (Alzheimer’s Disease Assessment Scale—Cognitive) [12,13,14,15] in cohabitants only. 

MMSE is the most commonly used test to screen for overall cognitive impairment and represents a rapid and reasonably sensitive tool for the quantification of cognitive abilities and their changes over time. It consists of 11 items divided into five sections that include verbal and non-verbal tests. The scores followed the attribution rules according to Spencer and Folstein (1985) [10] and were adjusted for age and education [11].

In the assessment of the cognitive status of cohabitants, the ADAS-Cog [12,13,14,15] was used in addition to the MMSE.

The broader ADAS scale is divided into two sections:

- ADAS-Non-Cog (non-cognitive) is used to describe a subject’s behavior problems;
- ADAS-Cog evaluates specific cognitive sufficiency characteristics sensitive to the process of deterioration from primary degenerative dementia.

The ADAS-Cog is particularly useful to determine the extent of cognitive decline and can help assess the degree of severity of dementia based on the person’s responses and scores.

It includes 11 tests—memory (immediate word recall, orientation, word recognition, remembering test instructions); language (Naming objects and fingers, commands, spoken language ability, Word-finding difficulty in spontaneous speech, Comprehension); praxia (Constructional praxis, Ideational praxis)

The total score on the ADAS-Cog ranges from 0 to 70. The final score is corrected for educational level.

Physical activity was evaluated in NCs only, based on ADL and IADL scales covering daily physical functioning, and more complex aspects of everyday life:

ADL (Activities of Daily Living) [16,17]; and

IADL (Instrumental Activities of Daily Living) [18];

In the group of cohabitants, the ADL and IADL scales were not performed because none of the subjects had functional autonomy deficits

The ADL was used to investigate the ability of the subject to carry out daily activities such as dressing, washing, and feeding independently. It involves the assignment of a point for each independent function so as to obtain a total performance score that ranges from 0 (complete dependence) to 6 (independence in all functions) [16,17].

The IADL (Instrumental activities of daily living), on the other hand, checks daily instrumental skills such as knowing how to count money. Also, for the calculation of the IADL index, a simplified scale is used, which provides for the assignment of a point for each independent function in order to obtain a total performance score that ranges from 0 (complete dependence) to 8 (independence in all functions) [18].

Finally, a global clinical judgment was also formulated by GPs using the following additional parameters—analysis of associated pathologies and clinical judgment for the presence/absence of dementia according to the guidelines of the Italian Society of Neurology [19].

### 2.5. Laboratory Blood Testing

The total oxidizing capacity of the serum was determined by testing the d-ROM (a reactive derivative of oxygen metabolites), whose chemical principle is based on the ability of a biological sample to oxidize N, N-diethyl para phenylenediamine (DPPD). The total antioxidant capacity of the serum was evaluated by the BAP (Biological Antioxidant Potential) test, which measures the ability of a sample serum to reduce iron from ferric to ferrous ionic form [20,21,22,23,24].

These tests were conducted using the Free Radical Elective Evaluator photometers (Diacron, Grosseto, Italy).

The APOE genotype was determined by a PCR-based method (Polymerase Chain Reaction) and subsequent restriction analysis [25].

### 2.6. Statistical Analysis

The main analysis of the data is descriptive in nature.

## 3. Results

We compared 29 NCs and 49 cohabitants aged 50–75 years, from 29 households. The characteristics of the study population are shown in Table 1.

### 3.1. Lifestyle Assessment

None of the NCs were regular smokers, compared to nearly one-third of the cohabitants. In contrast, NCs were significantly more likely to consume daily alcohol, with 77% drinking, on average at least one drink per day, compared to 48% of cohabitants. Of the subjects who regularly consumed alcohol, over 20% of the cohabitants had, on average >2 drinks daily, compared to only 5% of the NCs. Alcohol consumption by the NCs was almost exclusively red wine.

**Figure 1:**
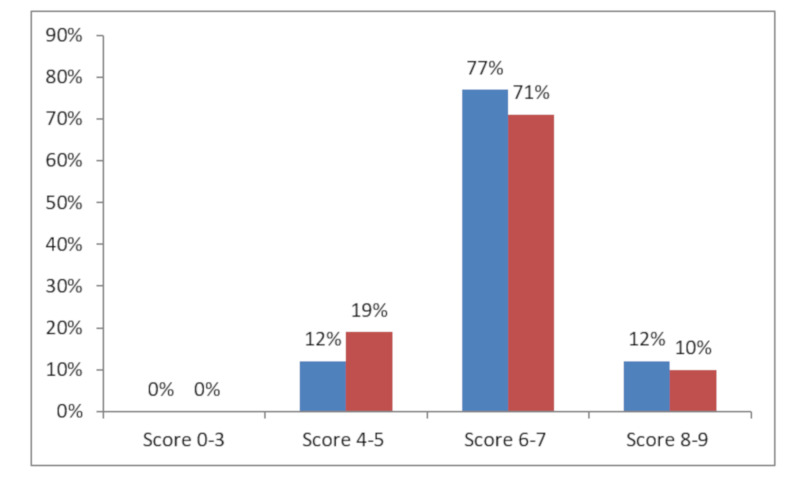
Mediterranean Diet Adherence Legend: score 0–3 = low; score 4–5 = intermediate; score 6–7 = satisfactory; score 8–9 = high Blue=NCs; red = Cohabitants.

With regard to the dietary lifestyle, the mean percent score of the adherence to the Mediterranean diet, evaluated through the MDS index, showed no meaningful numerical difference between the NC and cohabitant groups, with 89% and 81% of either satisfactory or high adherence, in NCs and cohabitants, respectively (Figure 1).

### 3.2. Cognitive and Neurological Assessment

The MMSE results are shown in Table 2. MMSE scores obtained in the NC group had values ≥24 in 16 subjects (55.2%), suggesting good overall cognitive functioning, and a score of ≤20 in 12 subjects (41%). One subject’s score was considered unreliable due to a severe hearing deficit and being illiterate. 

It is worth mentioning that the lowest MMSE score in NCs was 13. This seems to demonstrate that in such an elderly population, there was no subject with severe cognitive impairment on the MMSE scale—i.e., score below 10. 

In the final formulation of the diagnostic judgment, the relevance of tests administered and of the various associated pathologies was considered, but a relevant weight was also attributed to the clinical evaluation by the GPs. Their clinical evaluation allowed determination of the effects of these deficits on the autonomies of the subjects. Based on the careful analysis of this evidence in many of them, dementia was diagnosed in four out of 29 NCs. The results are summarized as follows:

2 (6.6%) subjects were affected by vascular dementia due to associated pathologies (stroke);

1 (3.3%) subject was diagnosed with degenerative dementia;

1 (3.3%) subject was diagnosed with mixed dementia.

The analysis of the data obtained from the ADAS-Cog scores in cohabitants (Table 3) demonstrated a very low score in 13 out of 49 subjects. The only tests of significant diagnostic value were the objective evidence of memory, i.e., the proof of "re-enactment of words" (subtest 1) in which 15 subjects had a high score, and the test of "recognition of words" (subtest 7) in which eight subjects had a high score. In total, only three subjects presented positive results for both subtests 1 and 7, two females and one male, and a diagnosis of Mild Cognitive Impairment (MCI) was then made in these three subjects. 

### 3.3. Functional Assessment

Data obtained on the ADL scale in the NC group (Table 4) showed that 16/29 of them were able to autonomously carry out daily life activities. The IADL scale analysis (Table 4) showed that 11 out of the 26 examined subjects were able to perform independent daily instrumental activities, while six were only partially able to perform them. There were no meaningful numerical differences in the scores between males and females.

The results from the analysis of the various scale data showed that in the 16 subjects with MMSE values ≥24, there were five subjects in whom the scores on ADL and IADL were low. For these subjects, the causes of lack of autonomy in the activities of daily life were due to problems of physical origin such as a bone fracture or serious impairment of the vision, as recorded in the initial clinical evaluation.

On the other hand, it was possible to obtain good scores on the ADL and IADL scales in NC subjects in whom the scores on the MMSE were rather low. This may be explained by the fact that the MMSE is an instrument that has limits, as noted above. 

### 3.4. Laboratory Tests 

The results of the comparative analysis of the individual data obtained in the NCs with dementia and MCI are summarized in Table 5 and Table 6. Looking at the three subjects in the cohabitant group with MCI and the relative NCs, the BAP values were lower than the normal value, and in all of the subjects with MCI, the values of d-ROMs were significantly higher than the normal value, in addition to three out of four NCs.

Oxidative stress Evaluation: in the NC group, only two subjects (6.9%) had normal values (above 2200 micromoles) on the BAP measure, nine subjects (31%) had a value lower than 2000, and 18 (62%) subjects had a value between 2000 and 2199 micromoles.

Four subjects (13.8%) had normal values (250–300 uCARR) of the d-ROMs measure, 21 subjects (72.4%) had a value greater than 350 uCARR, and four (13.8%) had a value between 300 and 350 uCARR. 

Only three (10.3%) subjects had an APOE4 positive tes and none of them had dementia.

In the cohabitants, only seven subjects (14.3%) had normal values (above 2200 micromoles) on the BAP measure, 13 subjects (26.5%) were below 2000, and 24 (49%) between 2000 and 2199 micromoles.

Five subjects (10.2%) had normal values (250–300 U.A.) on the d-ROMs measure, 32 subjects (65.3%) had values greater than 350 U.A., and 12 (24.5%) had values between 300 and 350 U.A. 

## 4. Discussion

Several studies have shown that centenarians’ family members have longer survival and are significantly healthier than other controls, thus representing a powerful, informative model for studying the origin of healthy aging and longevity [26,27,28,29,30,31]. Epidemiological data from different populations (American whites from New England, Mormons from Utah, Ashkenazi Jews residing in the United States, Icelanders, Japanese from Okinawa, and Dutch from Leiden) show how relatives (parents, siblings, and descendants) of long-lived people have significant survival advantage, an increased chance of being or becoming long-lived, have a lower risk of developing serious age-related diseases, such as cardiovascular and cerebrovascular diseases, diabetes, and cancer [32,33,34,35,36,37,38,39,40], and have a favorable lipoprotein profile [41,42]. Parents who later become NC probably adopt a healthier lifestyle for their children [43]. An interesting study of this type was conducted in the Italian population by Bucci [44]. In this study, functional status, instrumental skills, and cognitive status were assessed using the ADL, IADL, and MMSE scales, respectively. The reported measures as ADL and IADL were chosen since they have psychometric qualities that often do not provide equivalent information to physical tests, as suggested by recently published studies [45]. One of the strengths of this study is the experimental model, which includes the offspring of NC and the offspring of non-long-lived parents born in the same cohort of NCs. All participants were well studied, both at the general clinical level and at the molecular laboratory level. In our opinion, this is relevant since the aging process affects each organ of the body in a different way, giving rise to the so-called “Mosaic of aging” [46].

As the offsprings of NCs emerged as an excellent model for studying human aging and longevity, and longitudinal studies are needed to better understand the susceptibility and impact of diseases. Interestingly, in our study, the descendants and controls of the NC were recruited from the same geographical area, which makes it possible to assume that the two groups studied had similar eating lifestyles that may, therefore, be related to appearance, development, and impact of major age-related diseases. These food factors can also play a decisive role in the general health of the elderly, interfering with inflammation, oxidative stress, and the composition of the intestinal microbiota [46]. In Bucci’s study [44], in the group of centenarians’ family members compared to members of the non-centenarians ‘family, better scores on the functional and cognitive scales (ADL and MMSE) were highlighted. In our sample of members of the NC family, although not compared with members of the NC family, all members showed normal values on the MMSE, ADL, and IADL scales. This finding, therefore, shows similarities with the results obtained by Bucci et al. [44]. 

All over the world, NCs are generally women, and this aspect seems to be also confirmed in the Cilento population by our pilot study. In Italy, it is generally possible to identify a gradient of gender F:M from the north (7:1) to the south (3:1), while in the province of Nuoro in Sardinia, it becomes equal to 1:1 [47].

As expected, from the results of our study, it is possible to confirm that healthy habits were common in the NC group, including a lower prevalence of smoking compared to cohabitants and healthy eating habits. This is confirmed by the high adherence to the Mediterranean diet assessed by a nutritionist who went to the homes of the study participants to evaluate their daily food intake. These data also showed that a certain amount of daily red wine consumption seems to have a protective effect on the cognitive profile. In our study, the NCs indicated moderate alcohol consumption (one to two drinks per day) compared to their younger cohabitants, who were more likely not to drink alcohol at all or to drink more than two alcoholic drinks per day. NCs also tended to remain physically active with daily activities such as gardening and cooking and highly functional as confirmed by the ADL score, although they did not consider themselves as dedicated to regular exercise. A high score on the ADL scale was achieved in over 50% of NCs with substantial equality between the two sexes. On the IADL scale, most of the sample achieved satisfactory results, more women than men.

Alzheimer’s disease (AD) is the most common cause of progressive dementia, typically with onset after the age of 60. Studies of twins support the existence of a genetic form of the disease. Indeed, the only gene constantly found in the sporadic association, in various populations, is the apolipoprotein E4 gene (APOE4) [48,49,50,51,52,53,54,55,56]. 

Estimates on the prevalence of MCI are variable (from 3% to 42%), according to the definitions used [57]. In a meta-analysis of 41 cohort studies using MCI with a three-year follow-up, it was found that less than half of them were linked to the development of dementia [58]. In our study, the incidence of dementia was quite low in the NC population. Only in four subjects (13.2%) did doctors clinically diagnose dementia, including vascular dementia in two (6.6%), degenerative dementia in one (3.3%), and mixed dementia in one (3.3%). The measure we used for cognitive assessment was MMSE [10,11], the most commonly used tool for screening cognitive functions. MMSE is much more sensitive in detecting cognitive problems than informal questions and takes only about 10 minutes to administer, but is limited because it cannot detect memory loss, particularly in patients with a high level of education [12]. In subjects with low educational levels, this test could determine a low score even in the absence of cognitive deficit [26,27], while educated people can obtain a good score even in the presence of cognitive deficit [28]. MMSE provides guidance as a measure of retraction (immediate memory) and short-term memory (but not long-term memory) and language functions. MMSE may not be an appropriate test in patients with disabilities (e.g. sensory deficits) [10,59]. For this reason, in our study, the formulation of the final diagnosis of dementia took into consideration the MMSE, but also ADL and IADL scores in addition to the clinical judgment made by general practitioners, and showed a very low percentage of dementia in the group of NC (13.2%). In the group of cohabitants, using the administration of the ADAS-Cog scale, we were able to diagnose amnesic MCI in four subjects (7.8%), of which only two (3.9%) were children of NC. These subjects scored normal MMSE. The functional autonomy scales were not performed in any of the cohabitants because they were all autonomous.

In our sample, only three (10.3%) NC subjects and one cohabitant (8.2%) tested positive for APOE4. Of the three subjects in the NC group with APOE4, none had evidence of dementia, while in the four cohabitants with APOE4 had evidence of MCI [60].

Another aim of our study was to assess the blood oxidative stress, one of the recognized factors related to aging. Mitochondrial function produces reactive oxygen species (ROS) by dispersion of intermediates in the electron transport chain [61]. Usually, the harmful activity of a small percentage of these free radicals is neutralized by cellular, enzyme antioxidants (superoxide dismutase [SOD], catalase, glutathione peroxidase [GSHP]), and non-enzymes (vitamins E, C, and A). When, however, levels of free radicals increase (due to smoking, drug abuse, ultraviolet radiation, persistent chronic inflammation, etc.), the antioxidant pool is saturated, and excess free radicals damage biological structures. Oxidases and mitochondria are the main producers of ROS, which include highly reactive molecules such as superoxide anion. When the production of superoxide anion or H2O2 is so elevated to saturate the reductive capacity of SOD or GSHP, these molecules become substrates for the creation of highly reactive molecules such as hydroxyl (through a reaction of Fenton and Haber–Weiss) that are responsible for damage of cells and tissues [62]. The superoxide anion can react with nitric oxide in a limited reaction that generates peroxynitrite, in turn, a potent ROS. According to this hypothesis of longevity, the oxidation inflammation impacts on aging and healthy aging [61,62,63].

The only study that assessed oxidative stress in a NC population in Cilento [64] showed a slight increase for d-ROMs (324.1, SD 79.4) and values within limits for anti-ROMs 1 and 2 (234.4, SD 99.7 and 1188.8, SD 433.3), reflecting a good overall control of the oxidative balance. In that study [65], the NC population was divided into two groups based on the cognitive state, detected through the MMSE, showing that high d-ROMs was significantly higher in the group with cognitive impairment compared to the group with better cognitive performance. 

In the present study, BAP values were within the normal range in 7% and d-ROMs in 14% of NC cohort. On the other hand, in the cohabiting group, the BAP normal values were observed in 14% and d-ROMs in 10% of the subjects. These data suggest that the oxidative stress levels, particularly d-ROMs values, is unchanged with advancing age. This substantial uniformity of the values of BAP and d-ROMs (*p* = ns) between NCs and cohabitants appears to be of potential speculative interest as with increasing age, the oxidative damage is expected to increase, while the results in our sample indicate that probably different factors such as genetic substrate, adherence to a Mediterranean lifestyle (diet and psychosocial habits), microbiome, etc. could be involved in maintaining this balance unaltered over time, allowing for healthier aging. This observation is indirectly confirmed by results from other studies conducted in Cilento on oxidative stress and cognitive status where the d-ROMs values were found higher than the reference value (324 *vs*. 371) [65].

We believe that, in speculating on the reason for the good cognitive status of the long-lived Cilento population, it would be useful to refer to the paradigm of disability, disease, and death (DDD) [4]. This model suggests that the threshold at which DDD can be reached at different speeds in different individuals may be based on interactions between genes that play an important role in inflammation. The functional polymorphisms of Interleukin-6 and Interleukin-10 could be involved in the determination of the inflammation rate (slope), and therefore, of the point (age) where the DDD threshold is intercepted. Using this model in the interpretation of the data obtained in our population, it seems that the threshold of frailty/cognitive well-being can be influenced not only by genetic-inflammatory factors but also by a combination of other factors such as oxidation stress and lifestyle. In the future, it may be important to evaluate other parameters such as the microbiome, environmental determinants, specific nutrients, etc. in a larger NC population in different regions of the world. At the same time, other lifestyle factors such as physical, cognitive, and social activity, the consumption of red wine and positive personality traits such as the ability to recover, optimism, religiosity, and wisdom [7] may possibly contribute to health and longevity. Recent studies have shown that resilience and wisdom can be improved in the elderly through psychosocial interventions, resulting in a reduction of stress levels [64,66].

## 5. Conclusions

According to the results of this pilot study on a limited sample of NCs from the Cilento region in Southern Italy, NCs seem to have a good cognitive status without significant differences in the oxidative stress and the APOE genotype when compared to younger cohabitants aging 50–65 years. These results might be associated with optimal adherence to Mediterranean diet by the NC subjects, although the possible impact of other lifestyle factors as well as positive personality traits like resilience and wisdom on health and longevity should also be considered. Further studies on a larger population looking at more complex evaluations should be performed to confirm the results from this pilot study.

## Figures and Tables

**Table 1:** Subjects characteristics.

	Cohabitants (*N* = 49)	NCs (*N* = 29)
Age (years)mean +/− SD	61.67 +/− 5.52	93.69 +/− 3.34
% Female	53.1	62.1
MMSE mean +/− SD (NV 24–30)	26.08 +/− 1.56	21.80 +/− 4.33
ADL mean +/− SD (NV 4–6)	NT	4 +/− 2.07
IADL mean +/− SD (NV 6–8)	NT	3.38 +/− 2.27
BAP mean +/− (NV >2200 mcmol/L)	2088.92 +/− 129.98	2079.47 +/− 117.15
d-ROMs mean +/− SD (NV 200–300 uCARR)	371.16 +/− 65.85	386.17 +/− 65.85
APOE4 (% positive patients)	10.42	10.34
Lifestyle/Habits		
Current smoking (% of patients)	29.2	0
Daily Alcohol consumption (% of patients)	47.9	76.9
Regular physical activity (% of patients)	30.6	15.4

NCs: Nonagenarians and centenarians; NT = not tested; NS = not significant: SD = Standard deviation; MMSE = Mini-Mental State Examination; ADL = Activities of Daily Living; IADL = Instrumental Activities of Daily Living; d-ROM = reactive derivative of oxygen metabolites; BAP = Biological Antioxidant Potential; NV = normal value.

**Table 2:** MMSE (Mini-Mental State Examination) scores by age group and by sex.

Score	NCs			Cohabitants		
	Total (#)	Females (#)	Males (#)	Total (#)	Females (#)	Males (#)
	29	19	10	49	26	23
Unknown	1	0	1	-	-	-
0–10	0	0	0	0	0	0
10–19	12	8	4	0	0	0
20–23	0	0	0	1	1	0
24–30	16	11	5	48	25	23

# Stands for N.

**Table 3:** ADAS-Cog results in the cohabitants group; NT = not tested.

Score	Total of Subjects (#)	Females (#)	Males (#)
	49	26	23
NT	1	1	0
Subtest 1 (+)	15	7	8
Subtest 7 (+)	8	4	4
Subtest 1 e 7 (+) (−) Subtest 1 and/or 7	3 22	2 12	1 10

+: Patients positive and − for negative.

**Table 4:** ADL (Activities of Daily Living) and IADL (Instrumental Activities of Daily Living) Scores in NCs (Nonagenarians and centenarians).

Score	ADL			IADL		
	Total (#)	Females (#)	Males (#)	Total (#)	Females (#)	Males (#)
	29	19	10			
0–2	8	7	1	12	7	5
3–4	5	3	2	6	3	3
5–8	16	9	7	11	9	2

# Stands for N.

**Table 5:** Lab results in NCs with dementia and their cohabitants.

Patient Code	Age	Degree of Kinship	MMSE	ADAS-Cog	ADL	IADL	BAP (NV >2200 mcmol/L)	d-ROMs_Test (NV 200–300 uCARR)	APOE4 [Positive or Negative]
**1000**	**93**		**18.4**		**3**	**1**	**2115.00**	**376**	negative
1001	91	Sister	24.2		4	6	1910.72	354	negative
1002	54	grandson	27.2	3.2			1937.73	400	negative
1003	54	grandson	27.2	4.9			1964.71	366	negative
**2001**	**101**		**13.2**		**0**	**0**	**2166.00**	**346**	negative
2003	72	daughter-in-law	24.7	4.9			2148.53	454	negative
2002	74	son	24.7	9.3			2019.00	306	negative
**2081**	**95**		**15.2**		**5**	**2**	**2011.00**	**429**	negative
2082	67	Sister	25	5.3			2190.00	413	negative
2083	74	Son	25.3	3.6			2139.00	431	negative
**4011**	**91**		**17.2**		**6**	**1**	**1909.95**	**457**	negative
4012	56	Son	26.2	4.6			1885.65	266	negative
4013	57	daughter-in-law	28	3.2			2001.59	335	negative

Legend = data of subjects with dementia are in bold.

**Table 6:** Lab results in cohabitants with MCI and the NCs.

Patient Code	Age	Degree of Kinship	MMSE	ADAS-Cog	ADL	IADL	BAP (NV >2200 mcmol/L)	D_ROMS_Test (NV 200–300 uCARR)	APOE4 [Positive or Negative]
2091 PN	93		24.4		5	4	2165.00	471	negative
2092 MR	59	son-in-law	25.2	5.9			2176.00	345	negative
**2093 IM**	**61**	**daughter**	**27.2**	**6.9**			**1981.65**	**393**	**positive**
2111 AG	90		24.2		5	7	2109.15	425	negative
**2112 FS**	**62**	**son-in-law**	**28**	**3.6**			**2277.34**	**353**	**positive**
2113 MD	58	daughter	25.2	2.2			2282.40	310	negative
2061 AG	91		24.2		6	6	2174.00	273	negative
**2062 FI**	**64**	**son-in-law**	**25.9**	**11.6**			**2122.00**	**460**	**negative**
2063 EM	64	daughter	24.9	6.2			1947.65	448	negative
2031 EC	92		24.2		6	5	1985.86	484	positive
**2032 EC**	**60**	**son**	**24.9**	**3.3**			**2092.73**	**380**	**positive**
2033 TS	58	daughter-in-law	24.9	1.6			1994.39	335	negative

Legend = data of subjects with MCI are in bold.
